# Structural and school factors, affirmation and well-being among gender minority youth across Europe

**DOI:** 10.1093/eurpub/ckac129.679

**Published:** 2022-10-25

**Authors:** S Sevic, K Klasnic, DM Doyle

**Affiliations:** 1 Department of Sociology, University of Zagreb, Zagreb, Croatia; 2 Department of Psychology, University of Exeter, Exeter, UK

## Abstract

**Background:**

Previous research has shown that structural-level factors (discriminatory laws and policies) result in impaired health and well-being for transgender and gender diverse (TGD) adults. This study aims to assess if structural stigma is associated with school bullying/victimization and well-being among TGD youth and if more LGBTI supportive school environments, as well as social, physical, and legal gender identity affirmation ameliorate the effects of both structural stigma and experiencing violence.

**Methods:**

The study was conducted online in 2019 in 27 EU Member States and in the UK. We analyzed data from TGD students, aged 15 to 24 years, who at most had completed lower secondary education (n = 2,714). Well-being indicators used in the analyses included one-item measures of life satisfaction, depression, and considering leaving or changing school.

**Results:**

School environment, but not structural-level stigma, was associated with school bullying/victimization. Similarly, the effects of structural-level stigma, along with physical and legal gender identity affirmation were inconsistently related to students’ well-being across the multilevel models, whereas a more positive school environment and especially experiences of social identity affirmation were related to greater life satisfaction, better mental health, and lower odds of considering leaving or changing school; even though the negative effects of school bullying/victimization remained statistically significant across all models.

**Conclusions:**

This study's results suggest that compared with distal factors, more proximal factors - better school environment and social identity affirmation - have a greater impact on TGD students’ well-being. Given the inconsistency of our findings, more research is needed to understand the role of structural stigma and legal and physical gender identity affirmation in TGD students’ well-being.

